# Nonlinear temporal dynamics of cerebral small vessel disease: The RUN DMC study

**DOI:** 10.1212/WNL.0000000000004490

**Published:** 2017-10-10

**Authors:** Esther M.C. van Leijsen, Ingeborg W.M. van Uden, Mohsen Ghafoorian, Mayra I. Bergkamp, Valerie Lohner, Eline C.M. Kooijmans, Helena M. van der Holst, Anil M. Tuladhar, David G. Norris, Ewoud J. van Dijk, Loes C.A. Rutten-Jacobs, Bram Platel, Catharina J.M. Klijn, Frank-Erik de Leeuw

**Affiliations:** From the Donders Institute for Brain, Cognition and Behaviour, Centre for Cognitive Neuroscience, Department of Neurology (E.M.C.v.L., I.W.M.v.U., M.I.B., V.L., E.C.M.K., H.M.v.d.H., A.M.T., E.J.v.D., C.J.M.K., F.-E.d.L.), and Diagnostic Image Analysis Group, Department of Radiology and Nuclear Medicine (M.G., B.P.), Radboud University Medical Centre; Institute for Computing and Information Sciences (M.G.) and Donders Institute for Brain, Cognition and Behaviour, Centre for Cognitive Neuroimaging (D.G.N.), Radboud University, Nijmegen, the Netherlands; Department of Clinical Neurosciences, Neurology Unit (L.C.A.R.-J.), University of Cambridge, UK; and Erwin L. Hahn Institute for Magnetic Resonance Imaging (D.G.N.), University of Duisburg-Essen, Essen, Germany.

## Abstract

**Objective:**

To investigate the temporal dynamics of cerebral small vessel disease (SVD) by 3 consecutive assessments over a period of 9 years, distinguishing progression from regression.

**Methods:**

Changes in SVD markers of 276 participants of the Radboud University Nijmegen Diffusion Tensor and Magnetic Resonance Imaging Cohort (RUN DMC) cohort were assessed at 3 time points over 9 years. We assessed white matter hyperintensities (WMH) volume by semiautomatic segmentation and rated lacunes and microbleeds manually. We categorized baseline WMH severity as mild, moderate, or severe according to the modified Fazekas scale. We performed mixed-effects regression analysis including a quadratic term for increasing age.

**Results:**

Mean WMH progression over 9 years was 4.7 mL (0.54 mL/y; interquartile range 0.95–5.5 mL), 20.3% of patients had incident lacunes (2.3%/y), and 18.9% had incident microbleeds (2.2%/y). WMH volume declined in 9.4% of the participants during the first follow-up interval, but only for 1 participant (0.4%) throughout the whole follow-up. Lacunes disappeared in 3.6% and microbleeds in 5.7% of the participants. WMH progression accelerated over time: including a quadratic term for increasing age during follow-up significantly improved the model (*p* < 0.001). SVD progression was predominantly seen in participants with moderate to severe WMH at baseline compared to those with mild WMH (odds ratio [OR] 35.5, 95% confidence interval [CI] 15.8–80.0, *p* < 0.001 for WMH progression; OR 5.7, 95% CI 2.8–11.2, *p* < 0.001 for incident lacunes; and OR 2.9, 95% CI 1.4–5.9, *p* = 0.003 for incident microbleeds).

**Conclusions:**

SVD progression is nonlinear, accelerating over time, and a highly dynamic process, with progression interrupted by reduction in some, in a population that on average shows progression.

Markers of cerebral small vessel disease (SVD) are present on neuroimaging in virtually every individual over 60 years of age. They include white matter hyperintensities (WMH), lacunes, and cerebral microbleeds.^1^ SVD, and its progression, has been recognized as the most important vascular contributor to dementia.^2,3^ Therefore, understanding of the time course of SVD progression will result in better understanding of both etiology and consequences of SVD.

Current knowledge regarding temporal dynamics of SVD is limited due to lack of studies with more than one follow-up assessment. Consequently, these studies could only report the average, presumably linear change in SVD severity.^4–11^ Previous studies, however, have suggested that SVD progression may be a nonlinear process accelerating over time.^12,13^ Recent studies suggest that SVD might exert its clinical effects by affecting remote brain structure and function.^14,15^ The temporal relation between changes in SVD and the subsequent atrophy of these remote brain structures is thus far unknown.

Recently, decrease of WMH volume^12,13,16–18^ as well as decrease in number of lacunes^19,20^ and microbleeds^21,22^ have been reported, further challenging the assumption of linear progression of SVD markers. Neither the time course nor the magnitude of this disappearing SVD has been investigated.

In this study, we investigated the temporal dynamics of SVD by 3 consecutive neuroimaging assessments over a period of 9 years in participants with SVD, distinguishing progression from regression. As secondary analysis, we investigated the temporal dynamics related to atrophy of remote brain structures.

## METHODS

### Study population.

This study is part of the Radboud University Nijmegen Diffusion Tensor and Magnetic Resonance Imaging Cohort (RUN DMC) study, which prospectively investigates risk factors and clinical consequences of SVD. The detailed study protocol has been published previously.^23^ Of 503 baseline participants, 281 underwent repeated MRI assessment at 3 time points. Five participants were excluded because of insufficient scan quality, yielding a final sample of 276 participants for the present study (figure e-1 at Neurology.org).

### Standard protocol approvals, registrations, and patient consents.

The Medical Review Ethics Committee Region Arnhem-Nijmegen approved the study and all participants gave written informed consent.

### MRI protocol.

Images were acquired at 3 time points on 1.5T MRI (2006: Siemens [Munich, Germany], Magnetom Sonata; 2011 and 2015: Siemens, Magnetom Avanto) and included the following whole brain scans: 3D T1 magnetization-prepared rapid gradient echo (MPRAGE) imaging (voxel size 1.0 × 1.0 × 1.0 mm); fluid-attenuated inversion recovery (FLAIR) pulse sequences (baseline: voxel size 1.2 × 1.0 × 5.0 mm, interslice gap 1.0 mm; follow-up: voxel size 1.2 × 1.0 × 2.5 mm; interslice gap 0.5 mm); and a transversal T2*-weighted gradient echo sequence (voxel size 1.3 × 1.0 × 5.0 mm, interslice gap 1.0 mm). Full acquisition details have been described previously.^23^ The same head coil was used at all 3 time points.

To minimize effects of changes in FLAIR sequence, we resliced follow-up FLAIR images to match slice thickness of baseline images using linear interpolation.

### Brain volumetry.

Gray matter (GM), white matter (WM), and CSF probability maps were computed using SPM12 (fil.ion.ucl.ac.uk/spm/) unified segmentation routines on the T1 MPRAGE images. In addition, we used the WMH masks to correct the segmentation images, since several brain regions with WMH damage were initially misclassified. All WMH voxels were given mean WM intensity and these corrected T1 images were segmented using SPM12. All images were visually checked for coregistration and segmentation artefacts. GM volumes (GMV), WM volumes (WMV), and CSF volumes (CSFV) were computed by summing all voxels belonging to that tissue class multiplied by voxel volume in mL. Intracranial volume (ICV) was determined by summing GMV, WMV, and CSFV and total brain volume (TBV) by summing GMV and WMV.

To account for interscan effects, we corrected for differences in ICV between baseline and follow-up. We normalized all volumes to baseline ICV to account for head size.^24^

### Small vessel disease.

SVD was rated according to the Standards for Reporting Vascular Changes on Neuroimaging (STRIVE) criteria.^1^ WMH volumes were calculated by a semiautomatic WMH segmentation method, which has been described in detail elsewhere.^25^ Segmentations were visually checked for segmentation errors by one trained rater, blinded for clinical data. WMH volumes were corrected for interscan differences in ICV and then normalized to baseline ICV. We also calculated WMH volumes for odd and even slices separately to determine the effects of change in slice thickness of the FLAIR sequence.

We used the modified Fazekas scale to categorize WMH severity at baseline (mild: Fazekas 0–1; moderate: Fazekas 2; severe: Fazekas 3).^26^

Both number and location of lacunes and microbleeds were rated manually on FLAIR/T1-weighted and T2*-weighted MRI scans according to the STRIVE criteria^1^ by 2 trained raters blinded for clinical data. Follow-up FLAIR images were resliced to match the baseline scans to prevent differences in partial volume effects between baseline and follow-up scans. Interrater and intrarater reliability were excellent.^27^ Incidence was expressed as number of participants with new lacunes or microbleeds. We identified whether lacunes or microbleeds were truly incident or disappeared and in which time period. To minimize risk of misclassification due to coregistrations, we visually inspected all WMH segmentations and corrected lacune occurrence maps based on manual ratings.

### Vascular risk factors.

We assessed presence of hypertension, smoking, alcohol use, body mass index, diabetes, and hypercholesterolemia by standardized questionnaires, as described previously.^23^

### Statistical analysis.

We calculated differences in baseline characteristics between participants and those without follow-up using univariate analyses. Differences in Mini-Mental State Examination (MMSE) score between individuals with mild vs moderate or severe WMH at baseline and with or without WMH progression were examined using nonparametric tests.

We created WMH probability maps and distribution maps of lacunes. WMH decline was defined as more than 0.25 mL volume decline, as this was shown to be the smallest change that could be confirmed visually.^15^ We plotted change of WMH volumes by age at individual level using R package ggplot2 (version 2.1.0).^28^ R package lme4 was used to perform linear mixed-effects regression analysis to analyze WMH change as function of baseline age and time (version 1.1-12).^29^ We used a random intercept and random slope model, which permits the estimation of an average slope across the whole cohort while allowing for interindividual variability. By smoothed curves using loess smoothing we explored average WMH change with increasing age. To evaluate a possible quadratic relationship, indicating nonlinear progression of SVD, we compared model fit between the full model and the full model with a quadratic term for increasing age during follow-up included using a likelihood ratio test, and we evaluated change in Akaike information criterion (AIC).

To determine remote effects of SVD progression, we analyzed the relation between WMH progression in the first follow-up interval and subsequent brain atrophy by means of linear regression analysis. Multicollinearity between different SVD markers was investigated using regression analysis.

To identify differences in vascular risk factors in individuals with regression of SVD markers, we compared vascular risk factors for participants with and without regression of SVD markers and with those who remained relatively stable by analysis of variance followed by Bonferroni correction.

We created WMH probability maps stratified by baseline age and by baseline WMH severity. We repeated mixed-effects regression analysis stratified by baseline WMH severity to explore change in WMH within these groups separately. We calculated odds of SVD progression according to baseline Fazekas 0–1 vs Fazekas 2–3 by logistic regression analysis, adjusted for age and sex.

Statistical analyses were performed using SPSS Statistics version 20 and R Programming Language version 3.2.1.

## RESULTS

Baseline characteristics are presented in table 1. Mean age at baseline was 62.5 ± 7.7 years and 59.1% were male. Mean follow-up duration was 5.4 ± 0.2 years until first and 8.7 ± 0.2 years until second follow-up. Participants with moderate to severe WMH at baseline had lower MMSE scores (27.7 ± 1.7 vs 28.4 ± 1.5; *p* < 0.001) compared to participants with mild WMH. Steeper decline in MMSE score was seen in participants with WMH progression compared to participants whose WMH remained relatively stable (−0.95 ± 2.5 vs −0.32 ± 1.8; *p* = 0.031). Those lost to follow-up were significantly older and had more severe baseline SVD characteristics compared to participants (table e-1).

**Table 1:**
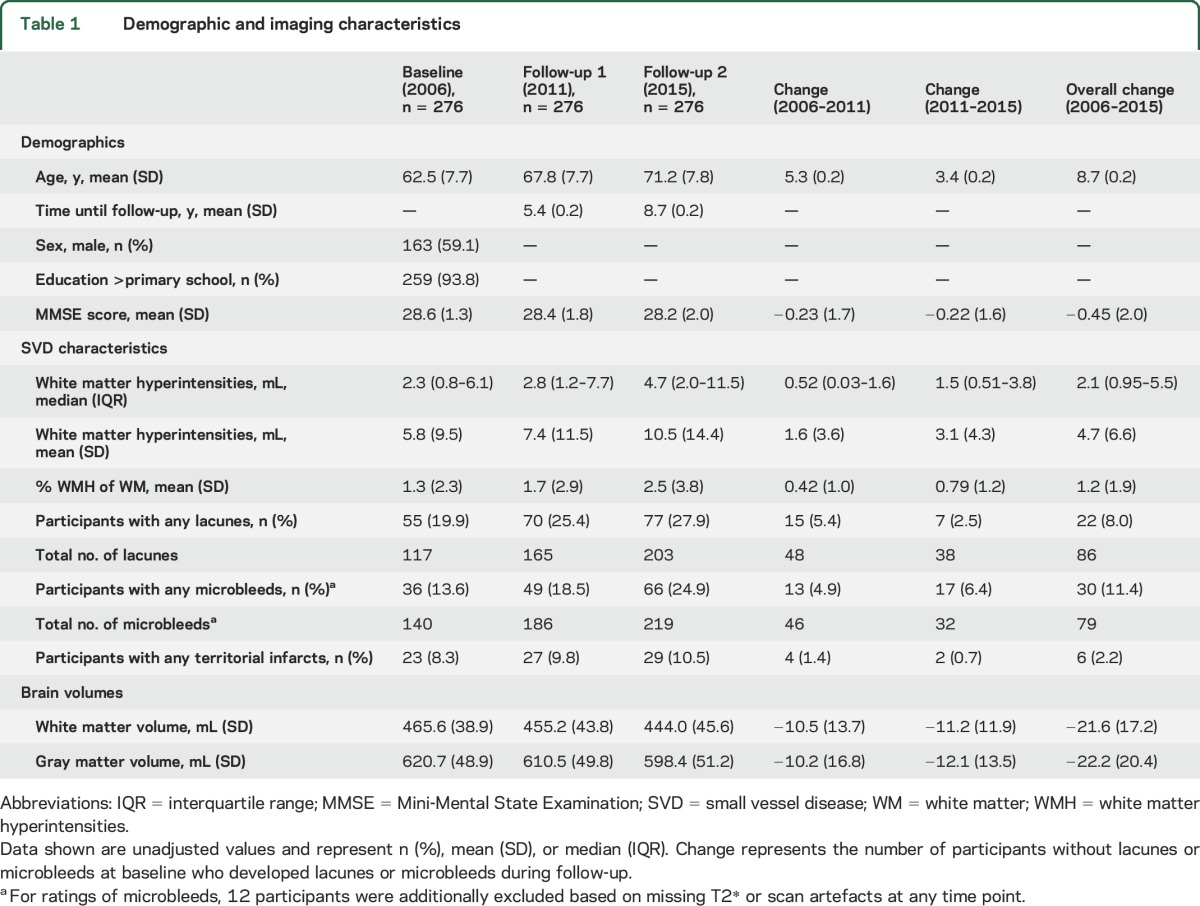
Demographic and imaging characteristics

### Temporal dynamics of SVD.

**Figure 1:**
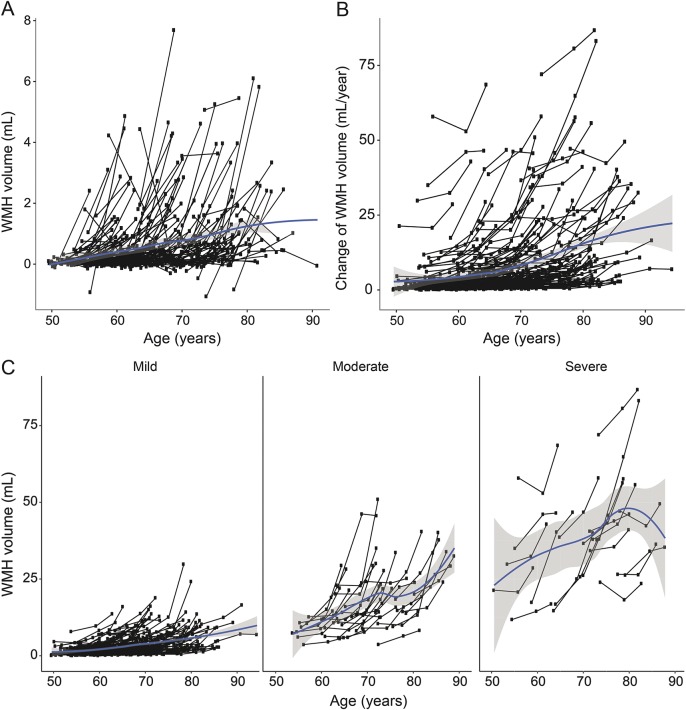
Temporal dynamics of white matter hyperintensities (WMH) progression (A) Change in WMH volume (mL) over 3 time points by age at individual level. (B) Acceleration of WMH volume change over 2 follow-up periods (mL/y) by age at individual level. (C) Change in WMH volume (mL) over 3 time points by age at individual level stratified by baseline WMH severity. Baseline WMH severity was classified as mild (Fazekas 0–1; n = 211), moderate (Fazekas 2; n = 33), or severe (Fazekas 3; n = 20). Smoothed curves using loess smoothing express average WMH change with increasing age.

WMH probability maps are shown in figure e-2 and video 1. Mean WMH progression was 0.54 mL/y (median 0.24; interquartile range [IQR] 0.11–0.64 mL/y). Progression of WMH increased with baseline age (figure 1, A and B; video 2). In mixed-effects regression analysis, each year increase of age at baseline resulted in an increase in WMH as percentage of WM of 0.10% (95% CI 0.07%–13.7%). Including a quadratic term for increasing age during follow-up significantly improved the model (AIC base model 2995.2 vs AIC extended model 2932.9, likelihood ratio test, χ^2^ = 64.3, *df* = 1, *p* < 0.001). Severity and progression of WMH were comparable for men and women.

Fifty-six participants (20.3%) developed new lacunes over 9 years (2.3%/y; table 2). The distribution of lacunes is shown in figure e-2 and video 3. Incidence of lacunes was higher for the second follow-up period (3.5%/y) than for the first follow-up period (2.7%/y).

**Table 2:**
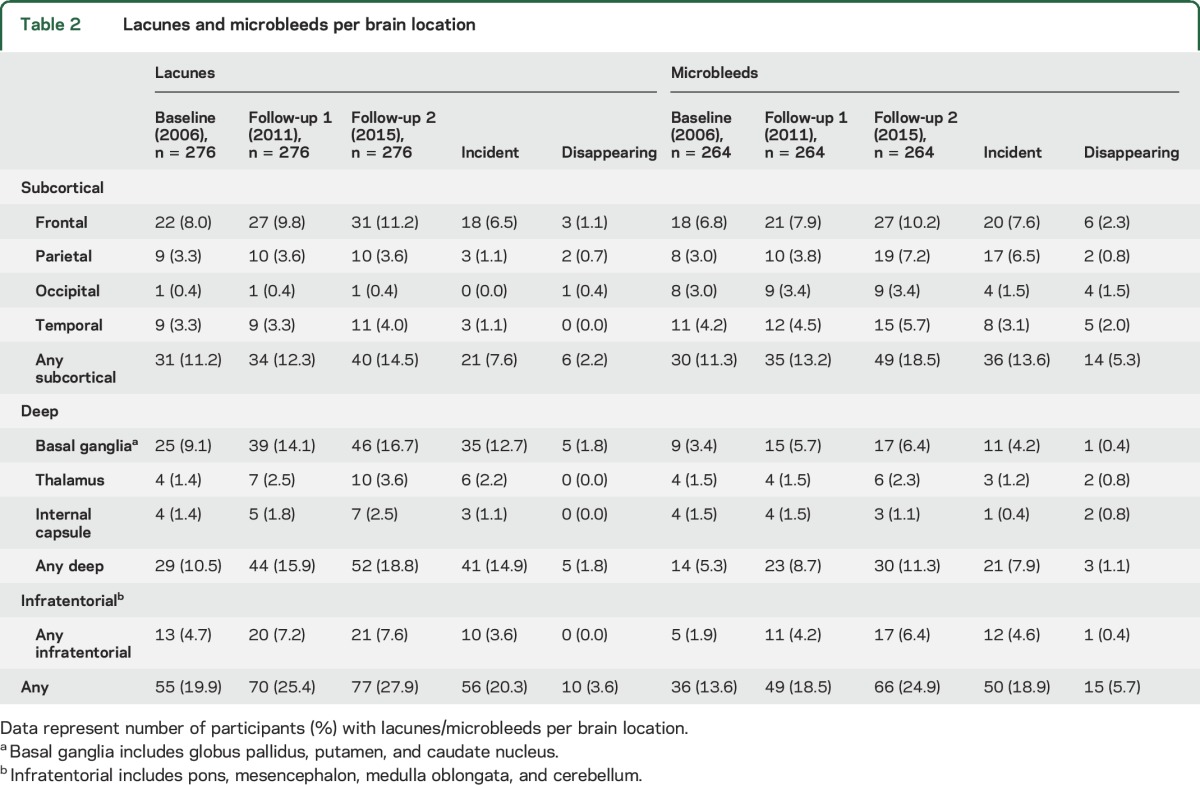
Lacunes and microbleeds per brain location

Fifty participants (18.9%) developed new microbleeds over 9 years (2.2%/y) (table 2). Incidence of microbleeds in the second follow-up period (4.2%/y) was higher than in the first follow-up period (1.7%/y). WMH progression in the first follow-up period was associated with brain atrophy in the second follow-up period (β = 0.124; *p* = 0.040) as well as with WM atrophy (β = 0.149; *p* = 0.013) but not with GM atrophy (β = 0.045; *p* = 0.461). Multicollinearity between SVD markers is shown in table e-2.

### Regression of SVD markers.

**Figure 2:**
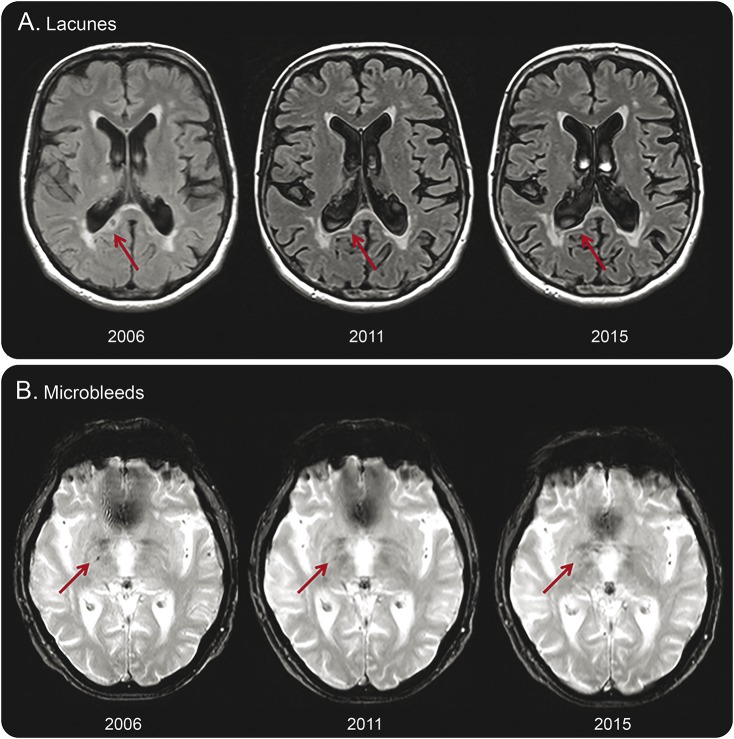
Lacunes and microbleeds no longer visible on follow-up imaging Examples of a lacune that is no longer detectable on follow-up imaging (A), which appears to be assimilated by the ventricle. Microbleeds (B) appear to have faded away over time.

We also observed regression of SVD markers. We observed decline in WMH volume in 26 participants (9.4%; median decline −0.5 mL; IQR −0.9 to −0.3 mL) during the first follow-up period and in 5 participants (1.8%; median −0.5 mL; IQR −0.9 to −0.4 mL) during the second follow-up period. In one participant, WMH volume declined over the course of 9 years (0.4%; −0.4 mL). In 10 participants (3.6%), 14 lacunes could not be found at follow-up imaging (table 2). In 15 participants (5.7%), 37 microbleeds were no longer detectable after 9 years of follow-up (table 2). Examples of lacunes and microbleeds that were no longer visible on follow-up imaging are shown in figure 2. There were no differences for any of the vascular risk factors between participants with and without regression of SVD markers, also compared with those who remained relatively stable over the 9-year course (data not shown).

### Heterogeneity in temporal dynamics of SVD.

**Figure 3:**
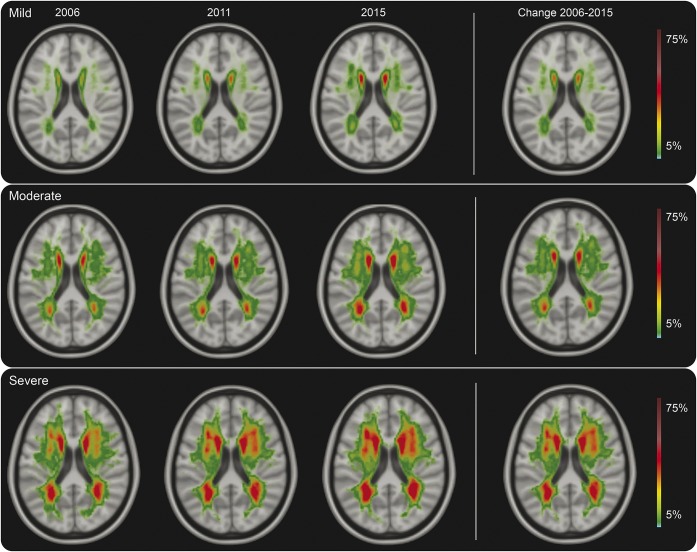
White matter hyperintensities (WMH) probability maps stratified by baseline WMH severity Probabilities of presence of WMH stratified by baseline WMH severity, color-coded in percentage from 5% to 75%. Baseline WMH severity is determined as mild (Fazekas 0–1; n = 211), moderate (Fazekas 2; n = 33), or severe (Fazekas 3; n = 20). The overall 9-year change is shown in the right column. Probability maps through the whole brain can be seen in video 4.

Mean WMH progression over 9 years was 2.4 mL for participants with mild WMH at baseline, 12.0 mL for those with moderate WMH, and 15.2 mL for those with severe WMH (figures 1C and 3 and video 4). From participants with mild WMH at baseline, 6% showed WMH progression beyond measurement error, compared with 75% of participants with moderate or severe WMH. Participants with moderate to severe WMH at baseline had 36 times higher risk of WMH progression compared to participants with mild WMH (OR 35.5, 95% CI 15.8–80.0; *p* < 0.001). Participants with moderate to severe WMH also more often developed incident lacunes (OR 5.7, 95% CI 2.8–11.2; *p* < 0.001) and microbleeds (OR 2.9, 95% CI 1.4–5.9; *p* = 0.003) compared to participants with mild WMH at baseline.

## DISCUSSION

In this study, we showed the temporal dynamics of SVD, revealing both SVD progression and regression, using 3 imaging assessments over a period of 9 years. We demonstrated that progression of all SVD markers occurred in a nonlinear fashion, accelerating over time consistent with a quadratic course. In addition, we showed that participants with moderate or severe WMH had a high likelihood of progression of their SVD, whereas participants with mild baseline SVD showed mild progression over a period of 9 years.

Our study demonstrates that SVD progression is not linear but accelerates with increasing age. While the average progression in our study is comparable with other studies,^4–11^ the use of 3 imaging assessments allowed us to show that SVD progression accelerated over time, providing evidence for a nonlinear process.^12,13^ Moreover, our results suggest that a quadratic course of SVD progression over time is plausible, since including a quadratic term improved the model. Although we would need more than 3 time points to further study exponential functions, our study indicates nonlinear temporal dynamics of SVD progression. Our findings do not support the hypothesized ceiling effect in which WMH progression reaches a certain threshold at high age and high lesion volume,^2^ as we also saw WMH progression in those at high age and with high SVD lesion load.

The relation between WMH progression and subsequent WM atrophy and TBV atrophy suggests that SVD affects adjacent brain structures. WM atrophy might be the result of disconnected white matter tracts due to SVD, leading to axonal loss by anterograde or retrograde degeneration, and subsequently the loss of brain volume.^3,30^ The clinical observation that patients with similar SVD burden show heterogeneity in clinical symptoms might be explained by disconnection of WM tracts.

Imaging assessments at 3 time points also enabled us to identify regression of SVD markers followed by progression, in a cohort that on average showed progression. This observation provides further evidence that SVD does not gradually evolve but is a dynamic process, with progression interrupted by regression in some. Thus far, few other studies have reported a decline in WMH volume,^12,13,16,17^ possibly because WMH decline within a certain time window was compensated by WMH progression thereafter (or vice versa). Two imaging assessments do not allow disentangling of episodes with regression from those with progression.

The observed decline in WMH may have several explanations. First, WMH decline in the first follow-up period could be explained in part by partial volume effects caused by slight adjustments in FLAIR sequences between baseline and first follow-up. However, this is unlikely because WMH volumes calculated from even and odd slices were identical and because we also found WMH decline between the second and third MRI assessment. Second, different orientation of participants in the scanner might also partly explain disappearing SVD, especially for smaller lesions. In order to prevent this, we classified WMH regression as more than 0.25 mL volume decline. Third, recently developed WMH might represent areas of tissue edema. Reduction in tissue edema at a later stage could lead to reduced WMH volume.^16^ Fourth, improved control of vascular risk factors or factors influencing the blood–brain barrier might play a role by reducing WMH volume.^16,17^ Disappearance of lacunes could be due to partial volume effects, due to collapsing lacunes or to incorporation of the lacune into the ventricle (figure 2).^19,20^ Disappearing microbleeds may be explained by partial volume effects as well as by clearance of hemosiderin-containing macrophages.^22^ Our findings are in line with the latter hypothesis. In most cases, microbleeds seemed to fade away between 2006 and 2011 and were no longer visible in 2015.

All SVD markers at baseline were important predictors for SVD progression, in a nonlinear way and independent of age. Additional analyses on progression of SVD markers by distribution of microbleeds did not reveal significantly different progression for participants with strictly lobar compared to participants with deep microbleeds (data not shown), although this analysis might have been underpowered. Although we would require an even longer follow-up to exclude the possibility that all participants with mild baseline WMH will ultimately progress to severe WMH, our data show that even the oldest participants with mild baseline WMH rarely show progression over a time course of 9 years. This suggests different progression curves for participants with mild vs severe baseline WMH, implying heterogeneity in etiology of mild vs severe SVD. Small WMH volumes, representing punctuate WMH or small periventricular caps, probably consist of enlarged perivascular spaces and subependymal gliosis.^7^ On the contrary, confluent WMH represent a continuum of ischemic tissue damage, ranging from mild fiber loss to complete infarction, and may have a more malignant course in terms of cognitive deterioration. These different etiologies call upon a different diagnostic and therapeutic approach. The correlation between WMH severity and progression and MMSE score underlines the clinical relevance of our findings on interindividual variability in SVD progression.

Strengths of this study include the large cohort of participants with SVD and the long follow-up duration. Furthermore, imaging assessments at 3 time points allowed us to characterize change in SVD over time, including SVD regression. SVD was rated according to standardized procedures,^1^ minimizing risk of misclassification. Moreover, semiautomatic WMH quantification reduced risk of information bias.^2^ Furthermore, brain volumes were determined with the newest segmentation routines of SPM12 and corrected for segmentation errors using WMH masks. Finally, our study has high external validity for patients with SVD in a general neurology clinic.

A limitation of our study is change of MRI scanner between baseline and first follow-up. However, by taking into account the third MRI assessment, we are able to capture most of this possible bias. A slight adjustment in FLAIR sequence between baseline and first follow-up may have caused an overestimation of incident lacunes. However, we limited the possible negative effects by reslicing follow-up to baseline FLAIR images before rating lacunes. Besides, changes in signal characteristics of normal brain tissue and WMH might have led to artefactually higher rates of lesion development. However, we considered this unlikely, since we also observed regression of SVD markers from the second to third time period in a considerable proportion of participants. Further, due to low-resolution T2* sequences, we might have missed smaller microbleeds. However, since similar gradient echo sequences are applied for all time points, risk of misclassification will result in comparable systematic error for all time points. Inevitably, attrition bias may have occurred due to the very long-term follow-up, probably leading to an underestimation of progression of SVD, since those who dropped out were older and had more severe SVD.

Our study demonstrates that SVD progression is a nonlinear, dynamic, and highly variable process, predominantly seen in participants with moderate or severe WMH at baseline. Equally important, those with mild WMH rarely show progression over a 9-year course. Since SVD progression has been linked to cognitive decline and development of dementia, our findings on interindividual variability in SVD progression might be a major step forward in developing personalized treatment approaches. The finding that progression of SVD is sometimes interrupted by regression and that SVD progression occurs in a quadratic way and hence is not gradually, linearly progressive as was previously thought suggests a paradigm shift on how SVD processes should be considered. Future studies should elaborate on the clinical consequences of this nonlinear dynamic time course of SVD progression.

## Supplementary Material

Data Supplement

Video

## GLOSSARY

AIC: Akaike information criterion
CSFV: CSF volume
FLAIR: fluid-attenuated inversion recovery
GM: gray matter
GMV: gray matter volume
ICV: intracranial volume
IQR: interquartile range
MMSE: Mini-Mental State Examination
MPRAGE: magnetization-prepared rapid gradient echo
STRIVE: Standards for Reporting Vascular Changes on Neuroimaging
SVD: small vessel disease
TBV: total brain volume
WM: white matter
WMH: white matter hyperintensities
WMV: white matter volume
