# Supplementary material for: Nonlinear temporal dynamics of cerebral small vessel disease: The RUN DMC study
Source: Neurology. 2017 Oct 10;89(15):1569–77. doi: 10.1212/WNL.0000000000004490 (PMC5634663; doi:10.1212/WNL.0000000000004490)
Supplement: Data Supplement [file supp_WNL.0000000000004490_Supplemental_data.pdf]

Supplemental data

Figure e-1: Flowchart

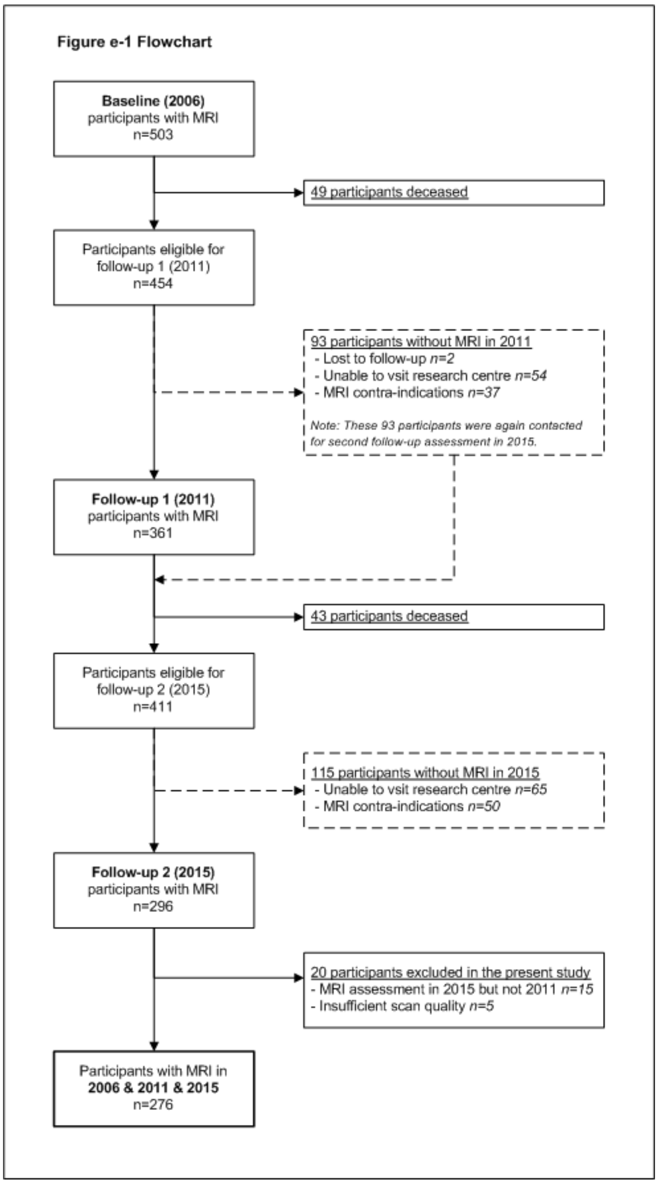

Design of the RUN DMC study. Imaging assessments were performed at three time-points over the course of nine years at baseline in 2006, at first follow-up in 2011 and at second follow-up in 2015. Note that the 93 participants who were unable to undergo first follow-up assessment in 2011 were again contacted for second follow-up assessment in 2015 (dotted lines). In total 281 participants underwent imaging assessments at all three time-points, of whom 276 participants were included in the present study.

Figure e-2: Probability maps of white matter hyperintensities and lacunes

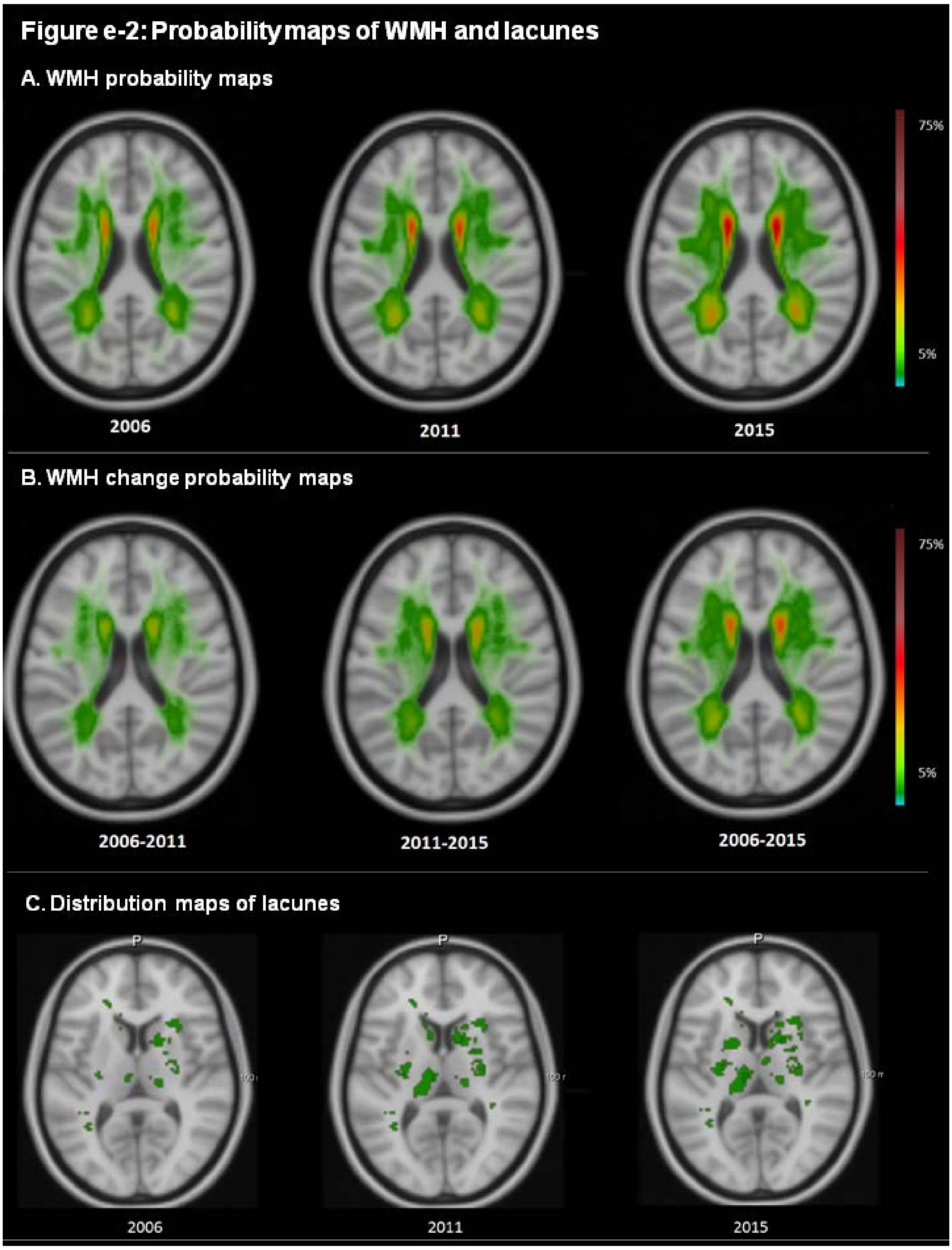

Probabilities of presence of WMH at three time-points (A) and probabilities to increase over these three time-points (B), colour-coded in percentage from 5 to 75%. Probability maps through the whole brain can be seen in **Video 1**. Panel C shows the distribution of lacunes at three time-points. Whole brain distribution maps can be seen in **Video 2**.

Table e-1: Baseline characteristics of participants compared with those lost to follow-up

	Participants n=276	Lost to follow up n=227	significance
**Demographics**			
Age (years), mean (SD)	62.5 (7.7)	69.5 (8.5)	**p<0.001**
Sex, male, no (%)	163 (59.1)	121 (53.3)	p=0.207
Education >primary school, no (%)	259 (93.8)	32 (14.1)	**p=0.004**
MMSE score, mean (SD)	28.6 (1.3)	27.6 (1.8)	**p<0.001**
**SVD Characteristics**			
White matter hyperintensities, ml, median (IQR)	2.3 (0.8-6.1)	7.7 (2.6-16.2)	**p<0.001**
White matter hyperintensities, ml, Mean (SD)	5.8 (9.5)	11.9 (13.6)	**p<0.001**
% WMH of WM, mean (SD)	1.3 (2.3)	2.9 (4.4)	**p<0.001**
Participants with any lacunes, n (%)	55 (19.9)	77 (33.9)	**p<0.001**
Total number of lacunes	117	135	**p=0.038**
Participants with any microbleeds, n (%) [o]	36 (13.1)	47 (20.9)	**p=0.022**
Total number of microbleeds [o]	140	159	p=0.496
Territorial Infarcts, n (%)	23 (8.3)	34 (15.0)	**p=0.023**
**Modified Fazekas score**			
Mild WMH (0-1), no (%)	218 (79.0)	114 (50.2)	**p<0.001**
Moderate WMH (2), no (%)	38 (13.8)	70 (30.8)	**p<0.001**
Severe WMH (3), no (%)	20 (7.2)	43 (18.9)	**p<0.001**
**Brain volumes**			
White matter volume, ml (SD)	465.6 (38.9)	441.5 (50.2)	**p<0.001**
Grey matter volume, ml (SD)	620.7 (48.9)	588.7 (51.6)	**p<0.001**
**Vascular risk factors**			
Smoking, ever, no (%)	196 (71.0)	157 (69.2)	p=0.696
Alcohol, glasses/week, mean (SD)	8.3 (9.0)	7.5 (9.7)	p=0.367
Glucose lowering drugs, no (%)	23 (8.3)	43 (18.9)	**p=0.001**
Hypertension, no (%)	190 (68.8)	179 (78.9)	**p=0.015**
BMI, mean (SD)	27.1 (4.1)	27.2 (4.2)	p=0.778
Lipid-lowering drugs, no (%)	118 (42.8)	119 (52.4)	**p=0.032**

Table e-1: Baseline characteristics of participants compared with those lost to follow-up

Data are represented as numbers (%), mean (SD) or median (IQR). Comparisons between participants and those lost to follow-up were performed by t-test, Chi-square or Mann-Whitney-U test.

[o] For ratings of microbleeds 4 participants were additional excluded based on missing T2*or scanartefacts at baseline.

Table e-2: Correlation matrix for baseline SVD characteristics and SVD progression

	Age	Sex	Baseline WMH volume	Baseline lacunes	Baseline microbleeds	Baseline WM volume	Baseline GM volume	Change WMH volume	Incident lacunes	Incident microbleeds	Change WM volume	Change GM volume
**Age**	…	…	…	…	…	…	…	…	…	…	…	…
**Sex**	-0.010	…	…	…	…	…	…	…	…	…	…	…
**Baseline WMH volume**	**0.315*** **	0.088	…	…	…	…	…	…	…	…	…	…
**Baseline lacunes**	**0.189** **	-0.102	**0.310*** **	…	…	…	…	…	…	…	…	…
**Baseline microbleeds**	**0.127* **	0.006	**0.222*** **	**0.237*** **	…	…	…	…	…	…	…	…
**Baseline WM volume**	**-0.460*** **	0.001	**-0.244*** **	**-0.205** **	**-0.132* **	…	…	…	…	…	…	…
**Baseline GM volume**	**-0.531*** **	**0.354*** **	**-0.258*** **	**-0.265*** **	**-0.161** **	**0.283*** **	…	…	…	…	…	…
**Change WMH volume**	**0.299*** **	0.101	**0.577*** **	**0.229*** **	0.110	**-0.182** **	**-0.281*** **	…	…	…	…	…
**Incident lacunes**	**0.137* **	-0.054	**0.299*** **	**0.447*** **	**0.129* **	**-0.165** **	**-0.151* **	**0.207** **	…	…	…	…
**Incident microbleeds**	**0.226*** **	0.049	**0.256*** **	**0.153* **	0.101	-0.104	**-0.147* **	**0.161** **	**0.173** **	…	…	…
**Change WM volume**	**-0.379*** **	**0.164** **	**-0.186** **	**-0.166** **	**-0.131* **	**0.202** **	**0.266*** **	**-0.295*** **	-0.085	**-0.172** **	…	…
**Change GM volume**	**-0.131* **	0.038	-0.036	-0.109	0.026	0.046	-0.093	-0.051	-0.112	-0.115	**0.189** **	…

Table e-2: Correlation matrix for baseline SVD characteristics and SVD progression

Correlations between baseline SVD characteristics and progression of SVD markers were determined by Spearman Rho for binary characteristics and by Pearson Correlation coefficients for continuous variables.
Correlations were significant at 2-tailed *p<0.05; **p<0.01; ***p<0.001.
