# Supplementary material for: Nonlinear temporal dynamics of cerebral small vessel disease: The RUN DMC study
Source: Neurology. 2017 Oct 10;89(15):1569–77. doi: 10.1212/WNL.0000000000004490 (PMC5634663; doi:10.1212/WNL.0000000000004490)
Supplement: Video [file supp_89_15_1569_v2_index.html]

Nonlinear temporal dynamics of cerebral small vessel disease — Video 

# Nonlinear temporal dynamics of cerebral small vessel disease

## Video

**Neurology® data supplements are not copyedited before publication. Published editorials and translations have been copyedited.  
 © 2017 American Academy of Neurology.  
  
 Files in this Data Supplement:**

- Video Legend - Microsoft Word file
- Video 1 - .mp4 file
- Video 2 - .mp4 file
- Video 3 - .mp4 file
- Video 4 - .mp4 file
