# Supplementary material for: Nonlinear temporal dynamics of cerebral small vessel disease: The RUN DMC study
Source: Neurology. 2017 Oct 10;89(15):1569–77. doi: 10.1212/WNL.0000000000004490 (PMC5634663; doi:10.1212/WNL.0000000000004490)
Supplement: Video [file supp_WNL.0000000000004490_Video_legends.docx]

**Video legends**

**Video 1: WMH probability maps**

Probability maps of WMH through the whole brain, colour-coded in percentage from 5 to 75%. This movie shows the probability of presence of WMH at three different time points (2006-2011-2015).

**Video 2: WMH probability maps stratified by baseline age**

Probability maps of WMH progression stratified by baseline age through the whole brain, colour-coded in percentage from 5 to 75%. This movie shows the WMH increase after 9 years of follow-up for participants aged <60 years, between 60 and 70 years and over 70 years.

**Video 3: Distribution maps of lacunes**

This movie shows the distribution map of presence of lacunes in three different time points (2006-2011-2015) in green, with incident lacunes in red.

**Video 4: WMH probability maps stratified by baseline WMH severity**

Probability maps of WMH progression stratified by baseline WMH severity through the whole brain, colour-coded in percentage from 5 to 75%. This movie shows the probability of WMH increase over 9 years of follow-up for participants with mild (Fazekas 0-1; n=211), moderate (Fazekas 2; n=33) and severe (Fazekas 3; n=20) WMH at baseline.
